# Exercises and the Blood-Pressure in Heart Cases

**Published:** 1910-07-30

**Authors:** 


### Medicine.

### EXERCISES AND THE BLOOD-PRESSURE IN HEART CASES.

In making researches on the blood-pressure after exercises it is important that controls should be made upon healthy subjects under the same circumstances as those under which the observations are being made upon the patients. The effects of exercises of varying degree upon heart cases undergoing the Nauheim treatment have been investigated by Dr. Hofmann. After a short rest in the horizontal position the blood-pressure is determined by Riva-Rocci's method before the exercises. Certain definite work is then done, and immediately afterwards the blood-pressure is tested in a resting position as before. The patient then does some more exercises, and at short intervals the blood-pressure is tested till the same pressure is shown as obtained before the gymnastics were begun. Great care must be taken that the Riva-Rocci manchette, or preferably the broad modification of it by Recklinghausen, is always applied in the same way, so as to eliminate as far as possible errors of variation.

The time during which these variations should be observed is about ten minutes. If the blood-pressure does not alter then we may rightly conclude that the exercises are doing the patient no harm and that they may be continued and increased. If it is raised it is not a bad sign if the rise be not too sudden. Even in healthy hearts we find the blood-pressure increased with exercise, but afterwards it falls very rapidly to normal. This is an important point in all cases of increase. A moderate rise and a quick return to the normal are favourable signs.

A lower blood-pressure after graduated work than before it is not necessarily a bad sign, provided it again rises quickly to the normal during rest. A fall after severe exercise may also be observed in healthy persons, and is brought about by the action of the nervus depressor causing dilatation of the cutaneous blood-vessels and so regulating the flow of blood from the aorta, where the pressure is highest; soon after exercise, however, the blood-pressure rises again to the normal, because during rest the increased activity of the heart diminished very rapidly, the high pressure in the aorta drops and simultaneously the action of the nervus depressor which has caused the dilatation of the cutaneous vascular system ceases. Thereupon the latter immediately contracts, the tranverse diameter of the blood-vessels becomes smaller and the blood-pressure rises, and may, without any bad results, even exceed the normal for a short time. It is different, however, if during rest after exercise the pressure remains for a long time below the normal. This indicates feeble working as a result of fatigue occurring in a weak heart. When the blood-pressure rises after exercises and remains above normal, we recognise in this continued secondary elevation Moritz's symptom of "strain and exhaustion" which indicates that too much has been done. We must also regard as a sign of strain and exhaustion the fall to below the normal of a blood-pressure which has risen to above the normal, and by the amount of rise, the extent of the fall, and the time taken to return to the normal, we form an estimate of the unfavourable effect and overstrain, which has resulted from doing too much. Finally, if the blood-pressure rises too violently and remains for a long time above the normal it indicates overstrain, after which we must always fear the effect of exhaustion.

